# Brain natriuretic peptide constitutively downregulates P2X3 receptors by controlling their phosphorylation state and membrane localization

**DOI:** 10.1186/s12990-015-0074-6

**Published:** 2015-11-14

**Authors:** Anna Marchenkova, Sandra Vilotti, Elsa Fabbretti, Andrea Nistri

**Affiliations:** Neuroscience Department, International School for Advanced Studies (SISSA), Via Bonomea 265, 34136 Trieste, Italy; Center for Biomedical Sciences and Engineering, University of Nova Gorica, 5000 Nova Gorica, Slovenia

**Keywords:** Trigeminal ganglia, ATP, Pain, Purinergic receptor, Purinergic signaling, Lipid raft, Protein kinase G (PKG), Sensory neuron

## Abstract

**Background:**

ATP-gated P2X3 receptors are important transducers of nociceptive stimuli and are almost exclusively expressed by sensory ganglion neurons. In mouse trigeminal ganglion (TG), P2X3 receptor function is unexpectedly enhanced by pharmacological block of natriuretic peptide receptor-A (NPR-A), outlining a potential inhibitory role of endogenous natriuretic peptides in nociception mediated by P2X3 receptors. Lack of change in P2X3 protein expression indicates a complex modulation whose mechanisms for downregulating P2X3 receptor function remain unclear.

**Results:**

To clarify this process in mouse TG cultures, we suppressed NPR-A signaling with either siRNA of the endogenous agonist BNP, or the NPR-A blocker anantin. Thus, we investigated changes in P2X3 receptor distribution in the lipid raft membrane compartment, their phosphorylation state, as well as their function with patch clamping. Delayed onset of P2X3 desensitization was one mechanism for the anantin-induced enhancement of P2X3 activity. Anantin application caused preferential P2X3 receptor redistribution to the lipid raft compartment and decreased P2X3 serine phosphorylation, two phenomena that were not interdependent. An inhibitor of cGMP-dependent protein kinase and siRNA-mediated knockdown of BNP mimicked the effect of anantin.

**Conclusions:**

We demonstrated that in mouse trigeminal neurons endogenous BNP acts on NPR-A receptors to determine constitutive depression of P2X3 receptor function. Tonic inhibition of P2X3 receptor activity by BNP/NPR-A/PKG pathways occurs via two distinct mechanisms: P2X3 serine phosphorylation and receptor redistribution to non-raft membrane compartments. This novel mechanism of receptor control might be a target for future studies aiming at decreasing dysregulated P2X3 receptor activity in chronic pain.

## Background

P2X3 receptors are trimeric cation channels gated by extracellular ATP, almost exclusively expressed by the majority of sensory ganglion neurons [1, 2], and important transducers of nociceptive stimuli [3, 4]. Even though the P2X3 receptor desensitizes rapidly (and, thus, self-limits its function), it can elicit fast, strong sensory neuron depolarization and firing which are actually enhanced in pathological pain states [4–8]. Certain endogenous modulators can upregulate P2X3 channels via multiple signaling pathways that alter their rate of synthesis, trafficking [9–11], phosphorylation state [10, 12–15], and receptor desensitization [10, 15, 16]. In particular, a major role is played by the neuropeptide calcitonin gene-related peptide (CGRP) that persistently enhances P2X3 receptor activity and, thereby, contributes to the development of algogenic syndromes in inflammation [17–19], chronic and neuropathic pain [10, 20], and migraine headache [21, 22]. It is, however, conceivable that endogenous substances may serve as negative regulators of P2X3 receptors under basal conditions. Their dysfunction might actually contribute to pain sensitization, a notion that could potentially paved the way to design novel analgesic drugs.

One candidate for the role of endogenous negative regulator of sensory ganglion activity is the brain natriuretic peptide (BNP). In fact, BNP downregulates inflammatory pain as well as firing frequency of small neurons in dorsal root ganglia (DRG; [9, 23]). BNP is one of the three structurally related paracrine factors belonging to natriuretic peptides family [24]. This peptide is a potent agonist on NPR-A receptors [24, 25] abundantly expressed by DRG CGRP-containing neurons [9] and trigeminal ganglion (TG) neurons [26] in which BNP-dependent NPR-A activation increases cGMP production [26, 27]. It is suggested that this system plays a constitutive inhibitory role in nociception mediated by P2X3 receptors because sustained pharmacological block of NPR-A strongly enhances P2X3 receptor mediated responses [26].

The molecular mechanism underlying the NPR-A dependent inhibition of TG P2X3 receptor function remains unclear. Our previous data indicate that NPR-A antagonism does not interfere with P2X3 expression [26], outlining a subtle process of P2X3 modulation. Potential targets might be the fine balance between phosphorylation and dephosphorylation controlling channel structure and function [3, 10, 13, 14, 28] or the differential distribution of P2X3 receptor between cholesterol-rich raft and non-raft membrane compartments [29–32].

The aims of the present study were to clarify the mechanisms and dynamics of BNP mediated constitutive regulation of P2X3 receptor function. Thus, we investigated changes in P2X3 receptor compartmentalization and phosphorylation state, and the role of certain intermediate steps in this process. To this end, BNP siRNA or the selective NPR-A blocker anantin were used to suppress NPR-A signaling and to evaluate any alteration in P2X3 receptor membrane distribution and phosphorylation state. Our results indicated multiple processes through which BNP-dependent NPR-A activity controlled P2X3 receptors.

## Results

### Unmasking tonic inhibition of P2X3 receptors by BNP

To support constitutive inhibition of P2X3 receptor activity by endogenous BNP in mouse TG neurons, the present study looked for changes in P2X3 receptor function after siRNA of BNP and compared this effect with the action of the pharmacological antagonist anantin.

**Fig. 1:**
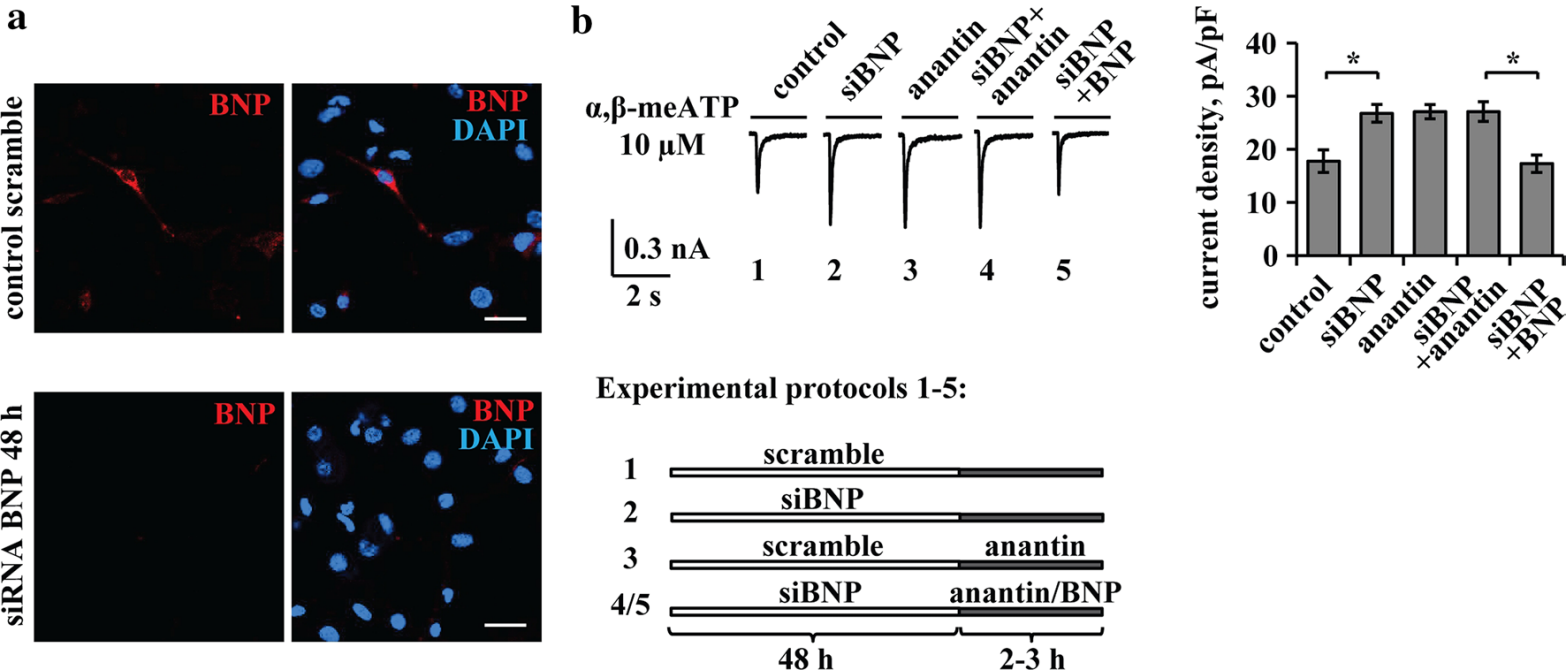
Effects of BNP siRNA. **a** Representative immunocytochemical examples of endogenous BNP expression in mouse TG cultures in control (*upper panels*) and after BNP siRNA (*lower panels*). Nuclei are visualized with DAPI (*blue*); *scale bar* = 20 µm. **b** Representative current traces show α,β-meATP (10 µM, 2 s)-evoked P2X3 responses in control, after 48 h of siBNP, anantin application (500 nM, 2–3 h), or after a combination of siBNP treatment with application of anantin or BNP (100 ng/ml, 2–3 h). The scheme at the *bottom* describes aforementioned experimental protocols. Histograms show average P2X3 current density values (n = 33, 33, 40, 32, 33, respectively; *p < 0.05, Kruskal–Wallis test). Note that effects of BNP silencing and anantin application are not additive and statistically indistinguishable; BNP applied after siBNP depresses P2X3 currents to the control level

Following BNP siRNA (48 h), no BNP (measured with the standard ELISA assay) could be detected in the culture medium, while in control conditions BNP was clearly detectable (4.7 ± 1.5 ng/ml), consistent with our previous report [26]. Furthermore, even if immunoreactive cells for BNP are just a few in cultured or in situ trigeminal ganglia ([26]; see top panels in Fig. 1a), after 48 h siRNA immunohistochemical staining did not demonstrate any BNP immunopositive cells in TG culture (Fig. 1a, bottom panels). P2X3 receptor mediated currents elicited by the selective P2X3 agonist α,β-methylene-ATP (α,β-meATP) [33] were next investigated using trigeminal sensory neurons (see experimental protocols schematized in Fig. 1b, bottom panels). Thus, BNP siRNA yielded larger amplitude responses that were similar to those recorded after anantin application (as exemplified in the records of Fig. 1b, left). When anantin was applied over a background of siRNA BNP, no further upregulation of P2X3 receptor function was observed, as seen from the histograms (Fig. 1b, right) and representative current traces (Fig. 1b, left). In addition, after siRNA BNP, 2 h application of BNP (100 mg/ml) depressed P2X3 receptor currents back to the untreated control level (Fig. 1b, right). These data, thus, validate that sustained block of NPR-A receptors with anantin and suppression of endogenous BNP synthesis evoked similar potentiation of P2X3 receptor activity. It is also noteworthy that a significant decrease in P2X3 receptor function was observed with BNP after siRNA, unmasking an inhibitory effect of this peptide that had not been previously detected.

### Dynamic modulation of P2X3 receptor function by the NPR-A blocker anantin

**Fig. 2:**
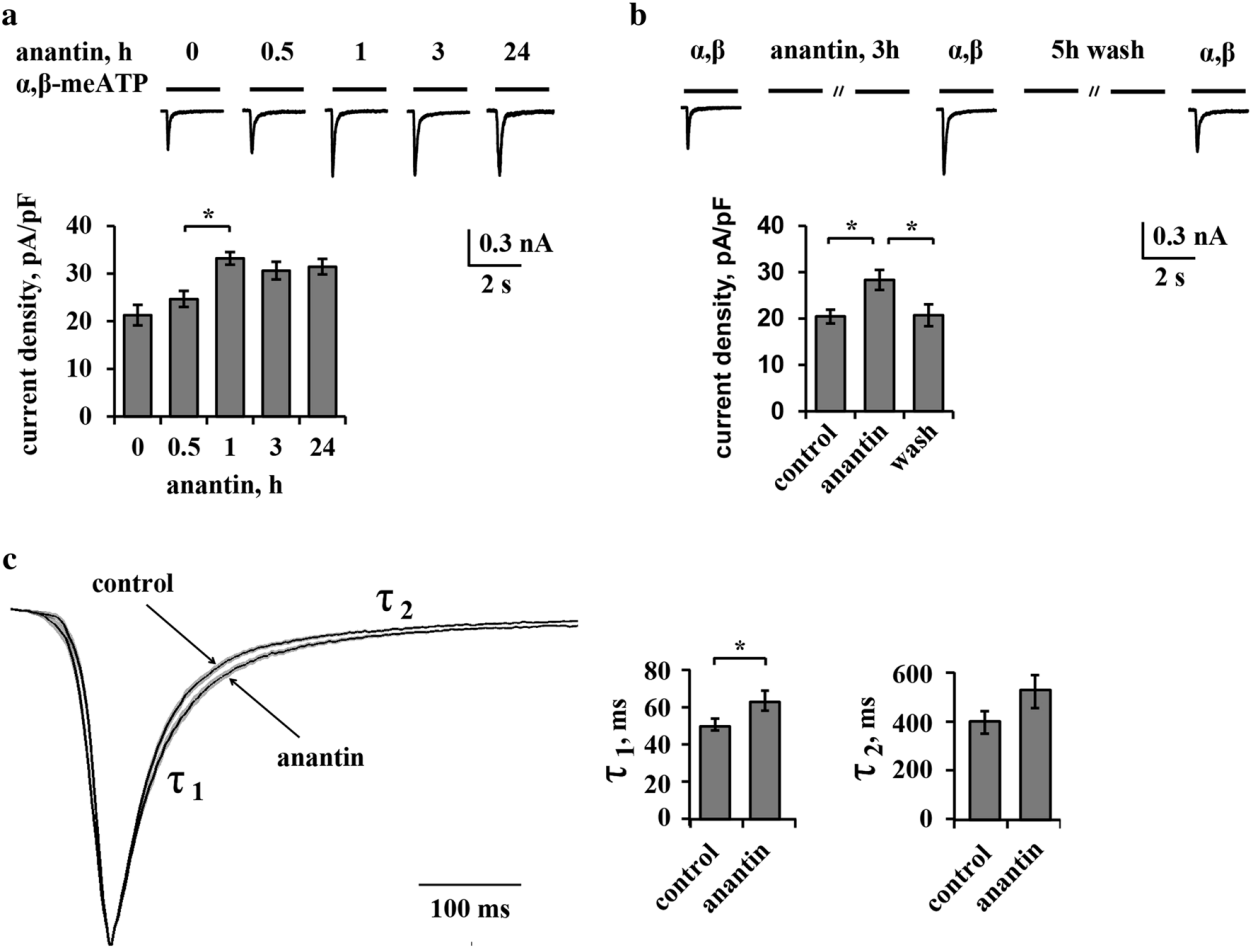
Time-course of anantin effects on P2X3 receptor currents. **a** Histograms together with representative P2X3 currents induced by α,β-meATP (10 µM, 2 s) show time-course of anantin (500 nM) effects on P2X3 currents. Anantin significantly increases mean P2X3 current density values after 1 h treatment already (n = 44, 30, 40, 40 for control, 1, 3 and 24 h treatment, respectively; *p < 0.05, Kruskal–Wallis test). **b** Representative traces obtained using α,β-meATP (10 µM, 2 s) show P2X3 current amplitudes in control, after 3 h anantin treatment (500 nM) and after 3 h anantin application followed by 5 h wash with standard physiological solution. Histograms show average P2X3 current density values in control, anantin and wash out conditions (n = 42, 40, 33, respectively; *p < 0.05, Kruskal–Wallis test). Note that 5 h wash abolishes anantin effects on P2X3 current density. **c** Superimposed average traces of scaled P2X3 currents (shown as mean; sem is within the trace thickness), in control and after anantin treatment (n = 67, 76, respectively, Mann–Whitney rank sum test). Note no significant change in the current onset and slowing down of the decay after anantin application; τ_1_, τ_2_ are the fast and slow components of P2X3 current desensitization dynamics. Histograms show mean values for τ_1_ and τ_2_ desensitization constants of P2X3 receptor currents in control and after 500 nM anantin application (n = 67, 76, respectively; *p < 0.05, Mann–Whitney rank sum test)

We next explored the timecourse of anantin potentiation of P2X3 activity, as this approach can cast light on the dynamics of the NPR-A modulatory action. Figure 2a shows examples of α,β-meATP (10 µM, 2 s)-induced P2X3 currents (Fig. 2a, upper panel) along with their mean current density values at various times of anantin treatment (Fig. 2a, lower panel). In fact, a significant increase in P2X3 currents was observed after 1 h of anantin (500 nM) application and remained at a stable plateau for longer (up to 24 h) exposures. Dose–response curves for α,β-meATP mediated currents in control and after 24 h exposure to 500 nM anantin were previously described [26], showing that this potentiation occurred throughout the agonist concentration range. Anantin (500 nM, 3 h) did not change cell input resistance (820 ± 57 MΩ in control versus 811 ± 58 MΩ after anantin treatment) or the baseline current (36 ± 5.7 pA in control versus 41 ± 9.1 pA after anantin treatment), indicating that anantin caused no significant change in background conductances that might have accounted for a broad, non-selective rise in neuronal membrane responses. We also studied whether the effect of anantin was reversible on washout: Fig. 2b illustrates that 5 h wash with standard physiological solution restored P2X3 current values to control level.

Because desensitization is an important process to limit P2X3 receptor-mediated responses [10, 15, 33], we investigated if anantin might have impaired P2X3 desensitization. Figure 2c, (left panel) shows averaged P2X3 currents (scaled and superimposed to aid comparison) in control solution or after anantin application (1 h). P2X3 currents normally decay with a bi-exponential time course [33], corresponding to the fast and slow components of P2X3 desensitization (τ_1_ and τ_2_; see also Fig. 2c, left) [34]. A total number of 161 currents was analyzed (85 in control and 76 after anantin application collected from several separate experiments) with peak amplitudes varying from 250 to 800 pA. Figure 2c shows a small, albeit significant, increase in τ_1_ values after anantin application compared to the control without statistically significant alteration in τ_2_ values (Fig. 2c, right; p < 0.05, Mann–Whitney rank sum test). Thus, retarding the onset of desensitization appeared to be one mechanism for the anantin-induced enhancement of P2X3 activity.

### Changes in extracellular CGRP do not affect anantin mediated P2X3 upregulation

**Fig. 3:**
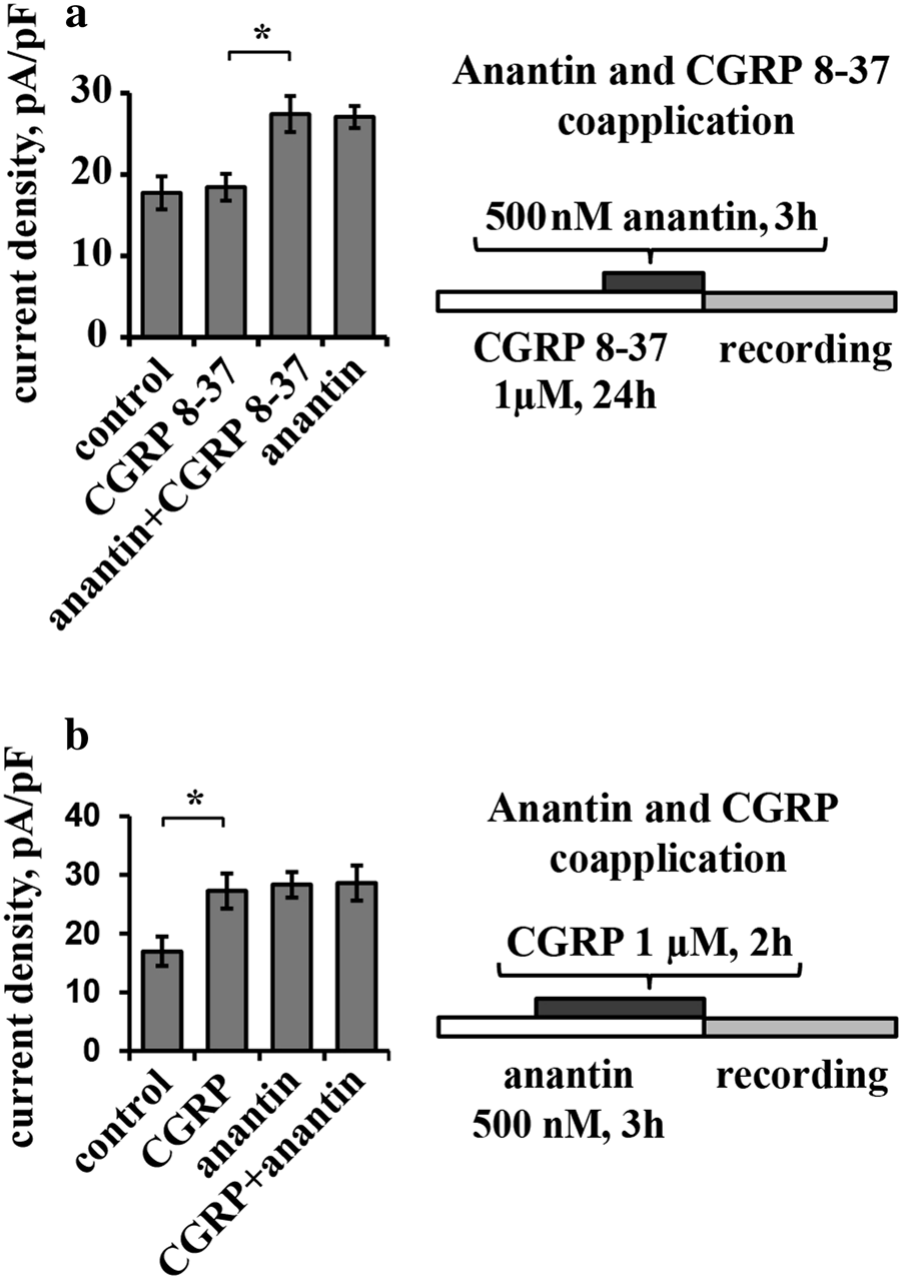
Anantin-mediated changes of P2X3 currents are not CGRP-dependent. **a** Histogram shows mean current density values of P2X3 receptors in control and after application of CGRP 8-37 (1 µM, overnight), anantin (500 nM, 3 h), or their combination (n = 44, 40, 50, 43, respectively; *p < 0.05, Kruskal–Wallis test). Pretreatment with CGRP 8-37 has no influence on anantin-mediated enhancement of P2X3 current density values, suggesting that this effect is not mediated via CGRP release. Scheme on the right describes experimental protocol of anantin and CGRP 8-37 coapplication. **b** Histograms compare mean current density values of P2X3 receptors in control and after application of CGRP (1 µM, 3 h), anantin (500 nM, 2 h) or their combination (n = 42, 40, 34, 33, respectively; *p < 0.05, Kruskal–Wallis test). Adding CGRP to the anantin treatment does not further increase P2X3 currents. Scheme on the *right* describes experimental protocol of anantin and CGRP co-application

CGRP is a powerful positive regulator of P2X3 function via complex signaling involving neurons and non-neuronal cells [28, 35–38]. We wondered if anantin might act by facilitating the action of ambient CGRP in trigeminal cultures [38]. Pretreatment with the selective CGRP receptor antagonist peptide CGRP 8-37 (1 µM, overnight; see the protocol scheme in Fig. 3a, right) per se did not modify P2X3 activity in accordance with our previous data [39] (Fig. 3a, left). Anantin retained its enhancing action on P2X3 currents even after CGRP 8-37 pretreatment (Fig. 3a, left).

We also studied whether the effects of CGRP and anantin were additive in upregulating P2X3 receptor currents. To address this issue, on the basis that the effect of anantin was manifested already at 1 h (Fig. 2a), we first applied this antagonist (500 nM) for 1 h and then together with CGRP (1 µM) for further 2 h when the action of CGRP is known to be reliably expressed [9]. With this protocol (Fig. 3b, right), the increase in P2X3 activity was the same as with either agent applied in isolation (histogram in Fig. 3b, left), indicating lack of additivity.

### Anantin promotes P2X3 localization to lipid rafts

**Fig. 4:**
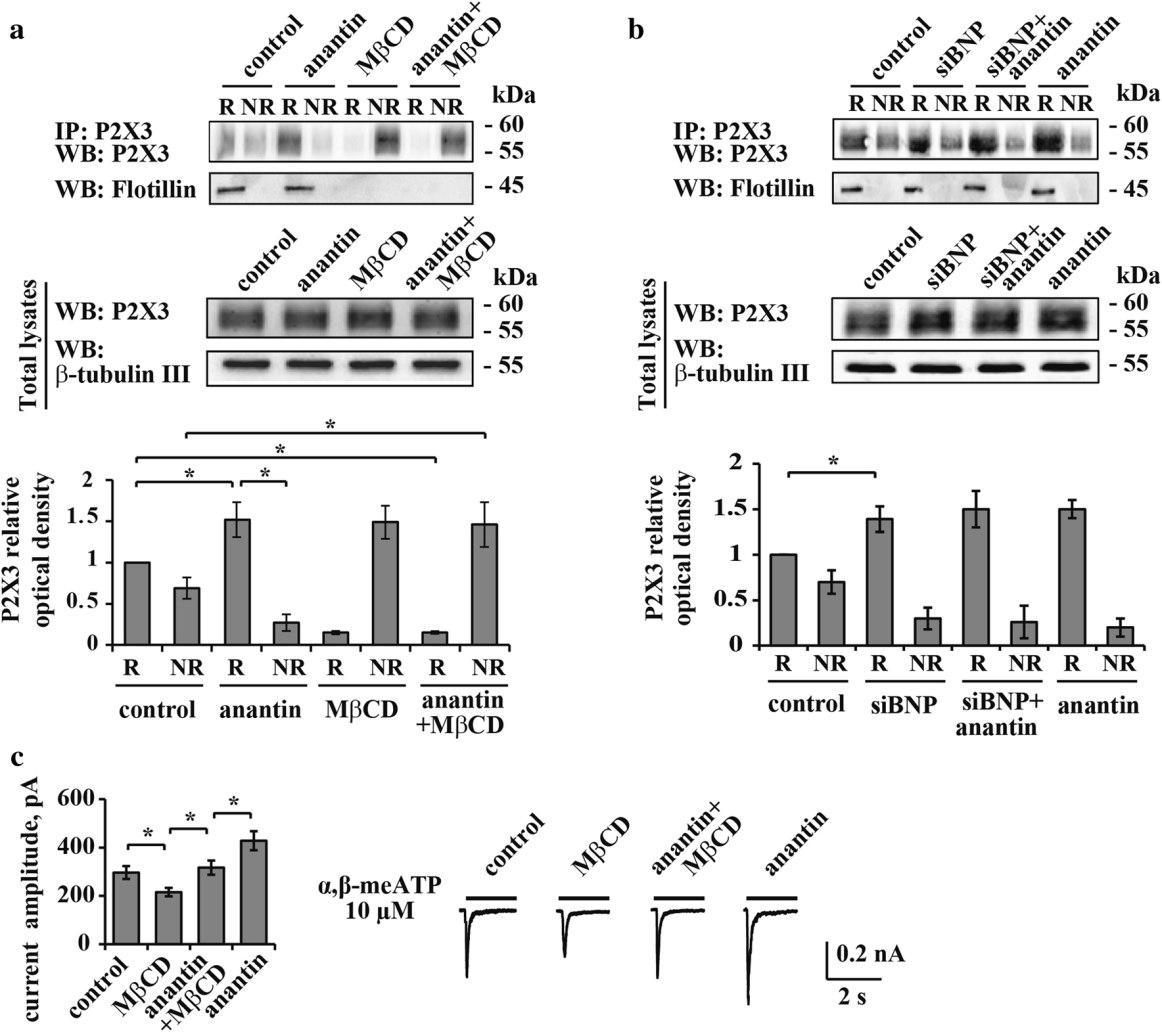
Anantin facilitates P2X3 localization to lipid rafts. **a** *Top panel* is a representative example of Western immunoblotting showing the amount of P2X3 receptors in raft (*R*) and non-raft (*NR*) membrane fractions in control and after treatment with anantin (500 nM, 24 h), MβCD (10 mM, 30 min) or their combination; flotillin bands indicate lipid raft membrane fractions. *Lower panel* shows total amount of P2X3 receptors for each experimental condition; β-tubulin was used as loading control of the total extract. Histograms at the bottom quantify mean P2X3 relative optical density values in lipid raft and non-raft membrane fractions (n = 4; *p < 0.05, Kruskal–Wallis test). Anantin relocates P2X3 receptors from non-raft to lipid raft membrane compartments, while MβCD abolishes anantin effect. **b** *Top panel* is a representative example of Western immunoblotting showing the amount of P2X3 receptors in raft and non-raft membrane fractions in control and after treatment with anantin (500 nM, 2–3 h), siRNA BNP (48 h), or their combination; flotillin bands indicate raft membrane fractions. Lower panel shows total amount of P2X3 receptors in each experimental condition; β-tubulin was used as loading control of the total extract. Histograms at the *bottom* quantify P2X3 relative optical density in lipid raft and non-raft membrane fractions according to the used treatment (n = 4; *p < 0.05, Kruskal–Wallis test). **c** Histograms show mean P2X3 current amplitude values in control and after application of MβCD (10 mM, 30 min), anantin (500 nM, 2–3 h), or their combination (n = 47, 58, 40, 61; *p < 0.05, Kruskal–Wallis test). Representative current traces of P2X3 receptors evoked by α,β-meATP (10 µM, 2 s) for each experimental condition are shown on the *right*. MβCD per se decreases P2X3 current density, whereas MβCD co-application with anantin returns current density values to control level

In previous experiments we observed how the differential localization of P2X3 receptors to lipid raft and non-raft membrane compartments of sensory neurons is an important process to characterize the efficiency of P2X3 signaling [29, 31, 32, 34]. Thus, using anantin we tested if the P2X3 receptor membrane distribution was dependent on the BNP/NPR-A system. In accordance with our former report [34], we obtained lipid raft (flotillin-labeled) and non-raft membrane preparations from control and treated cultures and tested them with Western immunoblotting as exemplified in Fig. 4a. Hence, a significant increase in the amount of immunopurified P2X3 receptors associated with the lipid raft compartment (R) vs the non-raft compartment (NR) was observed following 24 h anantin application (Fig. 4b, see examples in upper panel and data analysis at the bottom; middle panels show total lysates; n = 4, p < 0.05, Kruskal–Wallis test). It is noteworthy that, after anantin, there was no change in P2X3 receptor expression in total lysate samples that contained a broad assembly of membrane and intracellular P2X3 receptors with different degree of maturation (lower panel in Fig. 4a; see also [9, 12]). Consistent with previously reported data [34], the cholesterol-depleting agent MβCD (10 mM, 30 min treatment) disrupted lipid rafts almost completely (see loss of flotillin labelling in Fig. 4a, upper panel) and shifted a significant fraction of P2X3 receptors to the non-raft compartment, while the total amount of receptors remained constant (Fig. 4a, middle panel). Applying MβCD during the last 30 min of anantin administration blocked the anantin effect on P2X3 membrane distribution: thus, most P2X3 receptors were redistributed from lipid rafts to the non-raft membrane compartment (Fig. 4a, examples in upper panels and data analysis at the bottom).

Forty-eight hours after siBNP, we observed effects statistically indistinguishable from those of anantin per se in terms of localization of P2X3 receptors to the lipid raft membrane fraction (Fig. 4b, examples in upper panels and data analysis at the bottom). This phenomenon was unchanged by adding anantin during the last 3 h of silencing, while there was no alteration in the total lysate expression of P2X3 receptor protein (Fig. 4b, middle panels).

Electrophysiological recordings of α,β-meATP (10 µM, 2 s) induced P2X3 currents showed decreased peak amplitudes after MβCD treatment (p < 0.05, Fig. 4c, right), similar to previous data [34]. Data from this set of experiments are presented as peak current amplitudes and not as current density values, since MβCD treatment affects cell capacitance by depleting the cholesterol content of the cell membrane and, thus, its dielectric component (Fig. 4c, histograms on the left). After combined anantin and MβCD treatment, P2X3 current values were the same as in the control conditions (Fig. 4c, left and right). This result suggested that the combined administration enabled potentiation by anantin despite MβCD even though it could not reach the level observed with anantin alone (Fig. 4c, left and right).

### NPR-A receptors modulate P2X3 serine phosphorylation

Previous reports have indicated that the efficiency of P2X3 receptors depends on their phosphorylation state, concerning in particular their serine or tyrosine residues [12, 13]. To look for mechanisms through which the BNP system may modulate P2X3 function, we checked if anantin affected P2X3 serine or tyrosine phosphorylation by screening immunoprecipitated membrane P2X3 receptors with antibodies against the corresponding phosphorylated residues.

**Fig. 5:**
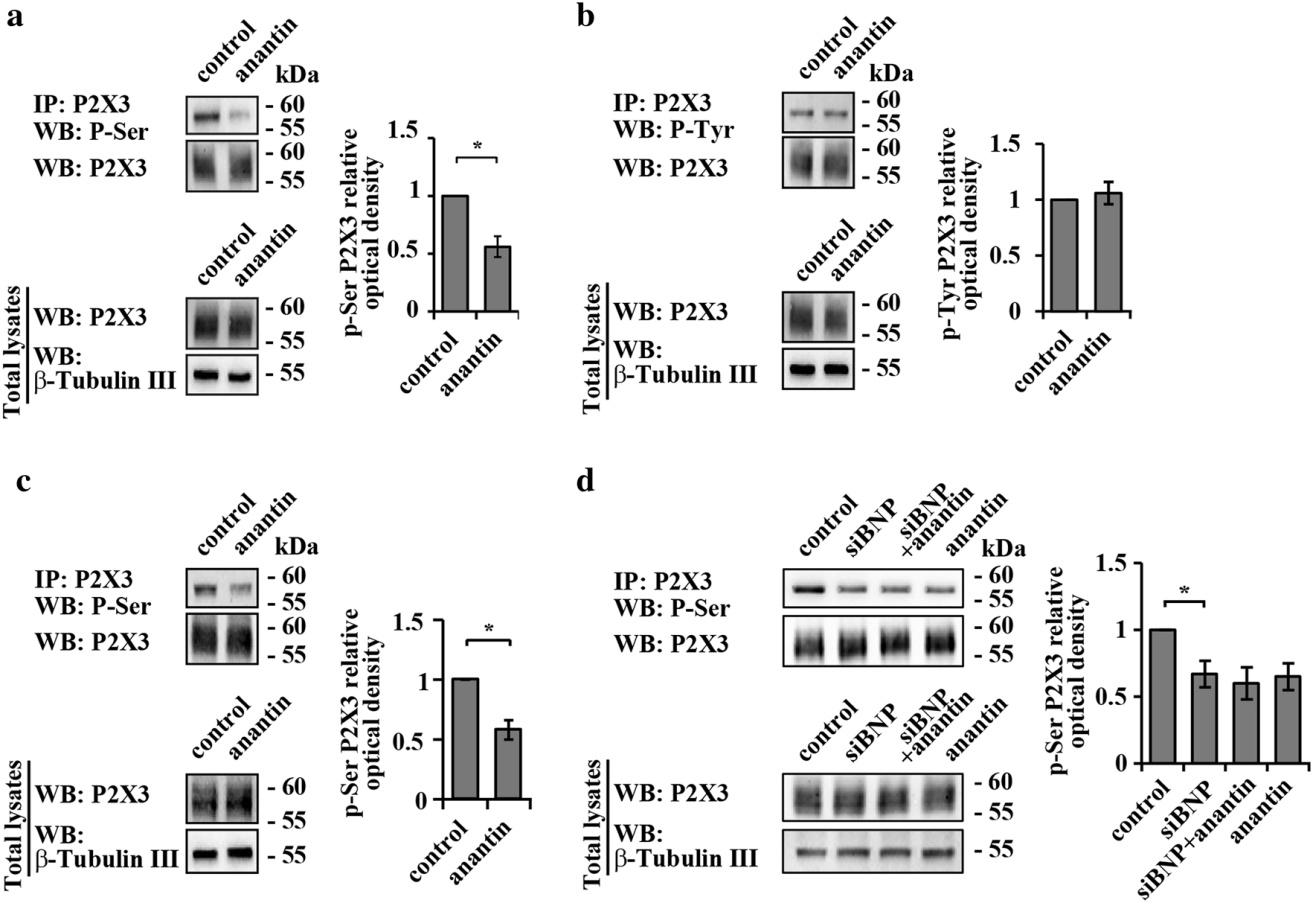
Anantin increases P2X3 serine phosphorylation. **a** Western immunoblotting shows the amount of P2X3 pSer and total amount of P2X3 receptors in control and after 1 h anantin (500 nM) application. β-tubulin was used as loading control of the total extract. Histograms on the *right* show statistically lower P2X3 pSer (relative optical density value) after anantin application compared to control (n = 4; *p < 0.05, Mann–Whitney rank sum test). **b** Representative example of Western immunoblotting, showing the amount of P2X3 pTyr and total amount of P2X3 receptors in control and after anantin application; β-tubulin used as loading control of the total extract. P2X3 pTyr does not change as shown in the plot on the *right*, summarizing pTyr P2X3 relative optical density in control and after anantin treatment. **c** Western immunoblotting shows the amount of P2X3 pSer and total amount of P2X3 receptors in control and after prolonged anantin application (500 nM, 24 h) (n = 3; *p < 0.05, Mann–Whitney rank sum test). β-tubulin used as loading control of the total extract. Note that anantin effects after 24 h or 1 h of treatment (panels **c** and **a**, respectively) are virtually indistinguishable. **d** Representative example of Western immunoblotting showing the amount of P2X3 pSer in control and after treatment with anantin (500 nM, 2–3 h), siRNA BNP (24 h), or their combination (n = 4); β-tubulin is used as loading control of the total extract. Histograms on the right quantify P2X3 pSer (relative optical density) according to the treatment (n = 4; *p < 0.05, Kruskal–Wallis test)

Figure 5a shows Western blot analysis of the serine phosphorylation (pSer) level in immunopurified membrane P2X3 receptors in control or anantin-treated cultures. The data revealed a significant reduction (n = 4 experiments, p < 0.05, Mann–Whitney rank sum test) of serine P2X3 phosphorylation (revealed as a single band; [13, 40]) following 1 h (Fig. 5a, examples in upper panels and data analysis on the right) or 24 h anantin application (Fig. 5c, examples in upper panels and data analysis on the right), in analogy to the upregulation of P2X3 currents (Fig. 2a). By comparison, P2X3 tyrosine phosphorylation (pTyr) remained unaltered (Fig. 5b).

Silencing BNP for 48 h (with or without later application of anantin) produced a reduction of P2X3 serine phosphorylation similar to the effect of anantin alone (Fig. 5d, examples in upper panels and data analysis on the right). Thus, inactivation of the NPR-A pathway by either blocking the receptors with anantin or exhausting cultures of endogenous BNP caused similar effects, confirming the idea of tonic regulation of P2X3 receptors activity by BNP via NPR-A.

Because calcineurin, CdK5, or CaMKII are known intracellular regulators of P2X3 receptor activity via changes in receptor phosphorylation [11, 13, 40, 41], we investigated whether blocking these enzymes might have effects analogous to those of anantin, and/or interact with the anantin potentiating action.

**Fig. 6:**
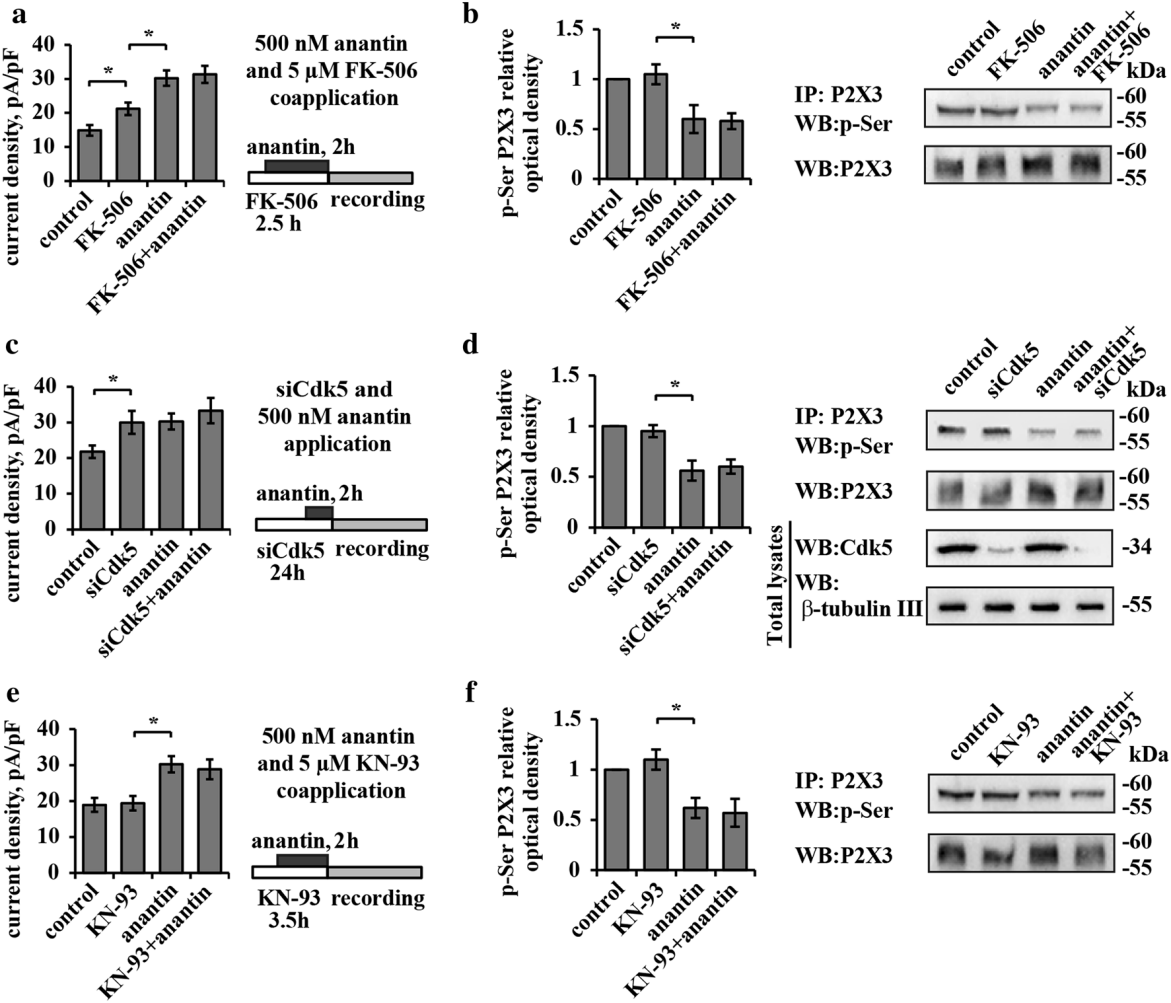
Anantin effects do not depend on the activity of CaMKII, Cdk5 kinase or the phosphatase calcineurin. **a** *Bar* plot summarizes means of P2X3 current density values in control and after application of calcineurin inhibitor FK-506 (5 µM, 2.5 h), anantin (500 nM, 2 h) or their combination (n = 37, 36, 40, 31, respectively; *p < 0.05, Kruskal–Wallis test). Scheme on the *right* describes experimental protocol of anantin and FK-506 coapplication. FK-506 pretreatment does not prevent anantin-induced increase of P2X3 current density values. **b** Representative example of Western immunoblotting (*right*) and the histogram (*left*), summarizing P2X3 pSer relative optical density values in control, after application of anantin (500 nM, 2 h), FK-506 (5 µM, 2.5 h), or their combination (n = 3; *p < 0.05, Kruskal–Wallis test). **c** Histograms show mean current density values of P2X3 receptors in control, after 24 h Cdk5 silencing with small interfering RNAs, after anantin application (500 nM, 3 h), or after a combination of those two treatments (n = 32, 31, 40, 30, respectively; *p < 0.05, Kruskal–Wallis test). Scheme on the *right* describes experimental protocol of combined siCdk5 and anantin treatments. Cdk5 RNA silencing does not prevent anantin from upregulating P2X3 receptor activity. **d** Representative example of Western immunoblotting (*right*) and histogram (*left*), summarizing P2X3 pSer (relative optical density values) in control, after application of anantin, 24 h siCdk5, or a combination of these treatments (n = 3; *p < 0.05, Kruskal–Wallis test); β-tubulin used as loading control of the total extract. **e** Histograms compare mean current density values of P2X3 receptors in control and after application of CaMKII inhibitor KN-93 (5 µM, 3.5 h), anantin (500 nM, 2 h), or their combination (n = 29, 26, 40, 28, respectively; *p < 0.05, Kruskal–Wallis test). Scheme on the *right* describes experimental protocol of anantin and KN-93 coapplication. KN-93 does not influence anantin-mediated increase in P2X3 current density. **f** Representative example of Western immunoblotting (*right*) and histograms (*left*), summarizing P2X3 pSer (relative optical density values) in control and after application of anantin (500 nM, 2 h), KN-93 (5 µM, 3.5 h), or KN-93 together with anantin (n = 3; *p < 0.05, Kruskal–Wallis test)

Figure 6a (left) shows mean values of P2X3 current density in cells treated for 30 min with the calcineurin inhibitor FK-506 (5 µM), that increased P2X3 currents without altering pSer P2X3, as previously reported [40]. Co-application of FK-506 together with anantin did not interfere with the effects of anantin on P2X3 receptors (Fig. 6a, b). Although Cdk5 is a negative modulator of P2X3 receptor activity [13], unchanged effects of anantin were also observed when we used 48 h Cdk5 siRNA treatment (Fig. 6c, d). Likewise, while CaMKII is reported to control P2X3 serine phosphorylation in TG neurons [40], the CaMKII antagonist KN-93 did not influence subsequent anantin effects on P2X3 receptors (Fig. 6e, f). In summary, the basal Ser phosphorylation of P2X3 receptors was under the distinct control of NPR-A receptors without apparently involving calcineurin, CdK5 or CaMKII.

### P2X3 serine phosphorylation and receptor membrane distribution

We next explored how the preferential lipid raft localization of P2X3 receptors and the decrease in P2X3 pSer were inter-related. Thus, we tested if lipid raft depletion with MβCD affected P2X3 pSer levels following anantin application.

**Fig. 7:**
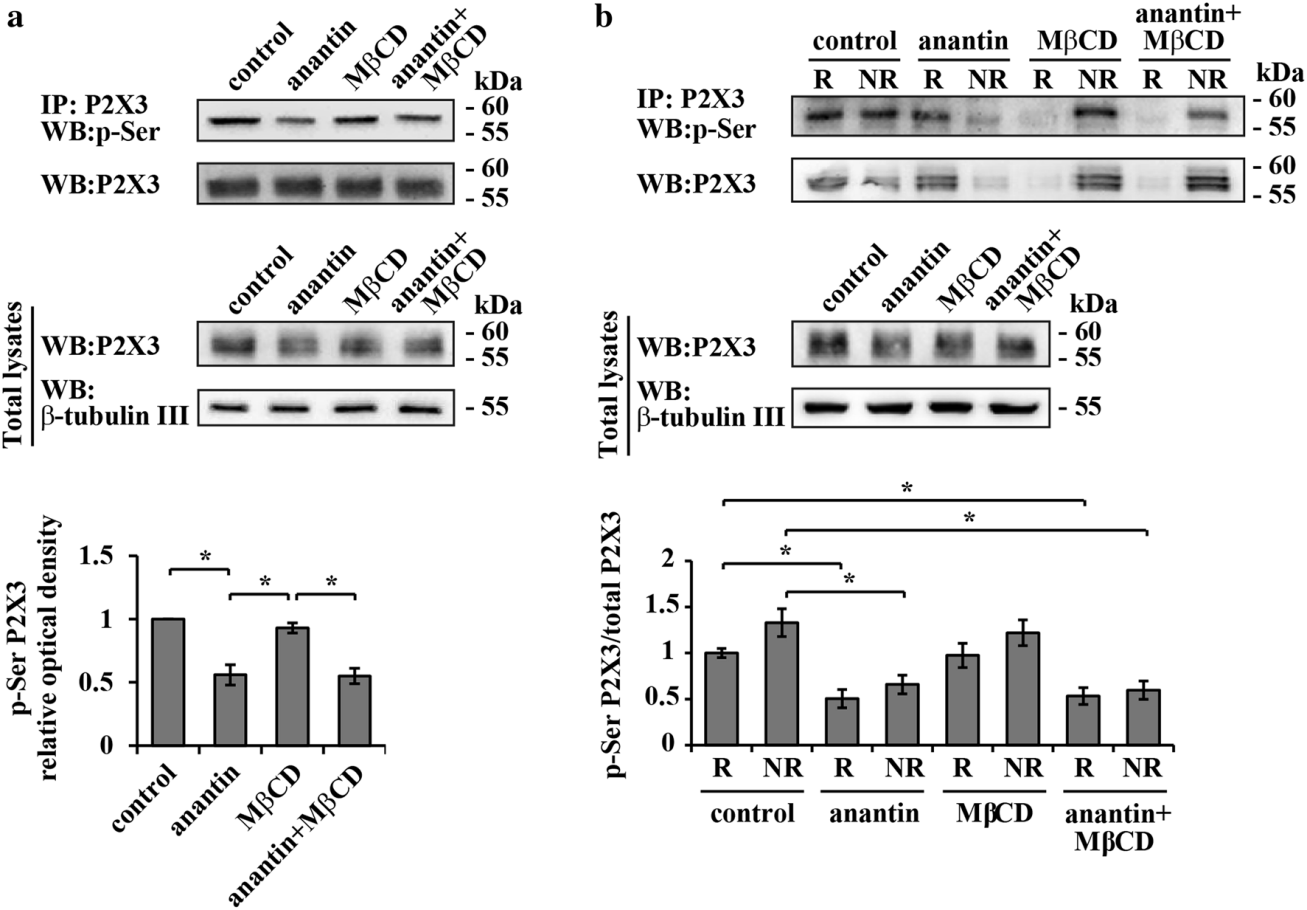
P2X3 serine phosphorylation in raft and non-raft membrane compartments. **a** *Top panel* shows representative example of Western immunoblotting with P2X3 pSer and anti-P2X3 receptor antibodies, summarizing the amount of P2X3 receptors in control and after application of anantin (500 nM, 2–3 h), MβCD (10 mM, 30 min), or their combination. *Lower panel* shows total P2X3 receptor amount for each experimental condition; β-tubulin used as loading control of the total extract. Histograms at the *bottom* quantify P2X3 pSer (relative optical density values) for each experimental condition (n = 4; *p < 0.05, Kruskal–Wallis test). Note that anantin effect on P2X3 pSer level is not influenced by MβCD. **b** *Top panel* shows representative example of Western immunoblotting with P2X3 pSer and P2X3 anti-P2X3 receptor antibodies, summarizing the amount of P2X3 receptors in lipid raft (*R*) and non-raft (*NR*) membrane compartments in control and after application of anantin (500 nM, 2–3 h), MβCD (10 mM, 30 min), or their combination. *Lower panel* shows total P2X3 receptor amount for each experimental condition; β-tubulin used as loading control of the total extract. Histograms at the *bottom* quantify the ratio between P2X3 pSer and total P2X3 in lipid raft and non-raft membrane fractions for each experimental condition (n = 4; *p < 0.05, Kruskal–Wallis test). Note that anantin reduces P2X3 pSer equally in both raft and non-raft membrane fractions and this effect is not influenced by MβCD

Figure 7a shows a representative example of Western blot analysis of P2X3 pSer levels in control or after application of anantin, MβCD or their combination. MβCD per se did not change basal P2X3 pSer level and did not influence the anantin-induced decrease in P2X3 pSer (Fig. 7a, upper panels and diagram at the bottom). Furthermore, there was no significant difference in the level of pSer of P2X3 receptors between raft and non-raft domains despite varying experimental conditions (Fig. 7b, upper panels and diagram at the bottom). Indeed, anantin produced an analogous two-fold reduction in P2X3 pSer in both compartments, regardless of the presence of MβCD.

Relying on these findings, it seems likely that P2X3 serine phosphorylation and receptor distribution between lipid rafts and non-raft membrane compartments were two separate processes targeted by the BNP/NPR-A pathway to regulate P2X3 receptor activity.

### NPR-A pathway is PKG dependent

Since NPR-A activation by BNP produces a rapid increase in cGMP level [25, 26, 42], one important downstream effector of this activity is the kinase PKG [9, 43–45]. We, therefore, inquired whether PKG might regulate P2X3 pSer and/or receptor distribution at membrane level.

**Fig. 8:**
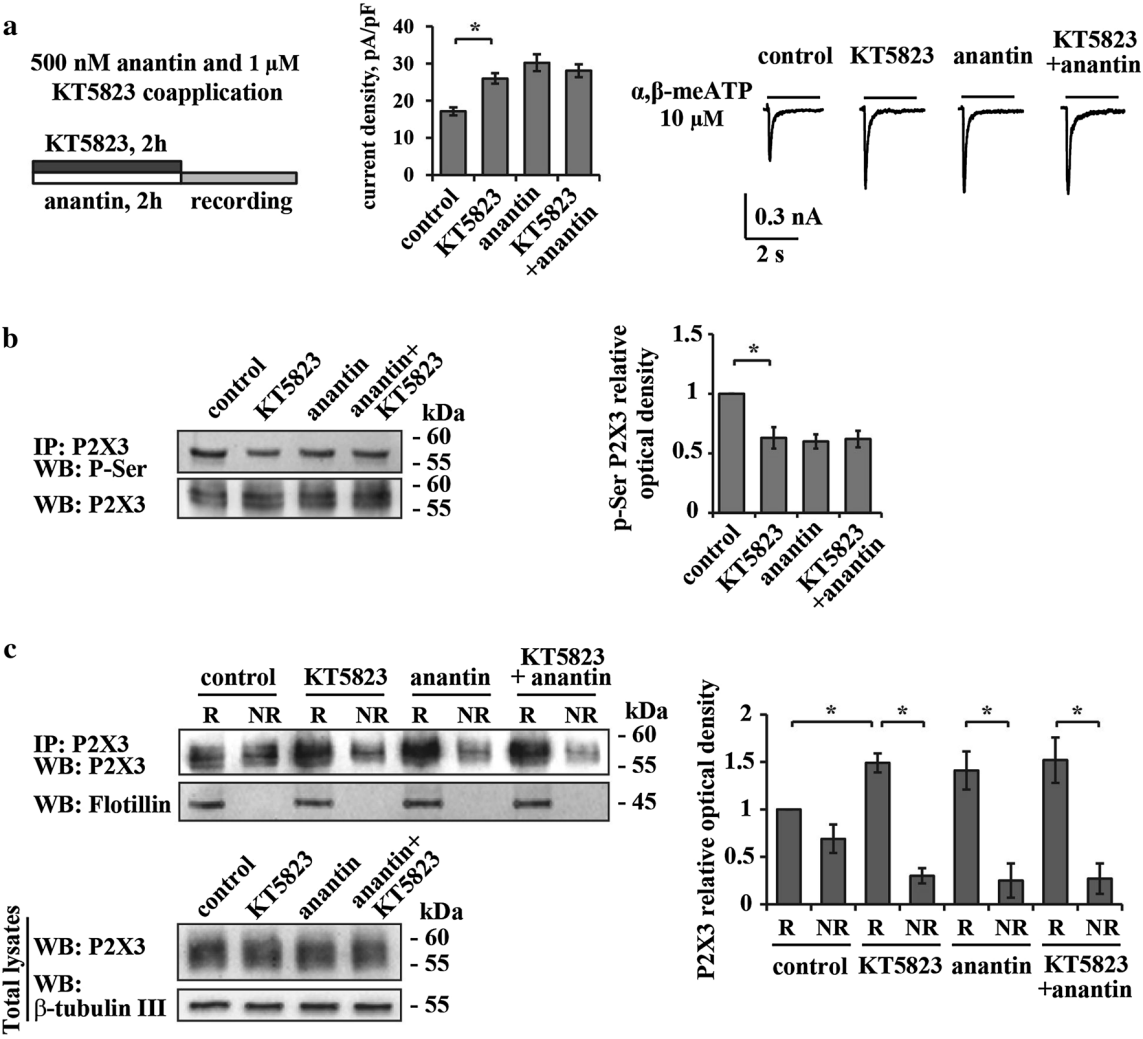
Effects of the PKG inhibitor KT 5823. **a** Scheme on the *left* describes the protocol of anantin (500 nM, 2 h) and the selective PKG inhibitor KT 5823 (1 µM, 2 h) co-application. Histograms (*middle*) show mean P2X3 current density values in control and after application of KT 5823 (1 µM, 2 h), anantin (500 nM, 2 h), or their combination (n = 33, 30, 40, 34, respectively; *p < 0.05, Kruskal–Wallis test). Representative current traces of P2X3 receptors for each experimental condition are shown on the right and were evoked by 2 s pulses of 10 µM α,β-meATP. KT 5823 produces anantin-like effect on P2X3 currents and shows no additivity when co-applied with anantin. **b** Representative example of Western immunoblotting with P2X3 pSer and P2X3 anti-P2X3 receptor antibodies (*left*) shows the amount of P2X3 receptors in control and after application of KT 5823 (1 µM, 2 h), anantin (500 nM, 2 h) or their combination; histogram on the right quantifies P2X3 pSer (relative optical density values) for each experimental condition (n = 3; *p < 0.05, Kruskal–Wallis test). Note that KT 5823 is as efficient as anantin in lowering the amount of P2X3 pSer. **c** Representative example of Western immunoblotting (on the *left*) and the histograms (on the *right*) summarize the amount of P2X3 receptors and P2X3 pSer in lipid rafts and non-raft membrane fractions in control and after application of KT 5823 (1 µM, 2 h), anantin (500 nM, 2–3 h), or their combination (n = 3; *p < 0.05, Kruskal–Wallis test). KT 5823 acts like anantin, causing P2X3 receptors redistribution to the lipid raft membrane compartment

Figure 8a shows that 2 h application of KT 5823 (1 µM), that is reported to be a selective PKG inhibitor at this concentration [46, 47], significantly increased control P2X3 current density values (Fig. 8a, middle and right), therefore mimicking the anantin action without any additive effect when co-applied with it. Likewise, KT 5823 produced anantin-like effects on both P2X3 phosphorylation (Fig. 8b) and receptor distribution to raft/non raft membrane fractions (Fig. 8c), with no additivity when co-applied together with anantin. Hence, it is suggested that PKG, operating as an early gateway of the BNP/NPR-A mediated molecular cascade, regulated P2X3 pSer and receptor distribution within the membrane compartments.

## Discussion

The novel result of the present study is the demonstration that constitutive activation of NPR-A by endogenous BNP inhibited P2X3 receptor activity in mouse trigeminal neurons. This process required two distinct modulatory mechanisms: P2X3 serine phosphorylation and receptor distribution to non-raft membrane compartments. This is the first observation of an intrinsic process that may functionally restrain inappropriate activation of pain-sensing P2X3 receptors, and it raises the possibility that chronic pain, at least at the level of the trigeminal territory, might originate from dysfunction of endogenous negative regulators.

### Negative control by BNP over P2X3 receptors

A difficulty in demonstrating this role of NPR-A receptors (and of its ligand BNP) was the experimental need to block them to reveal their contribution to P2X3 receptor activity. Thus, one important finding of the present work is that, once background BNP had been depleted by siRNA, we could demonstrate that exogenous BNP inhibited P2X3 function. Because depletion of BNP by siRNA had a basal facilitatory effect on P2X3 receptors, we propose that NPR-A receptors needed background levels of BNP to exert their effect rather than being simply activated even in the absence of their natural ligand. Consistent with that notion, the ambient concentration of BNP in TG cultures was rather low, yet clearly detectable even in the extracellular medium [26] and apparently sufficient to regulate P2X3 receptors. Furthermore, the effects of siBNP seem to suggest that BNP itself rather than other related natriuretic peptides was responsible for the modulation of P2X3 receptors. Our interpretation is that, since basal levels of BNP constitutively depressed P2X3 receptors, unmasking this phenomenon required blocking BNP or its receptor. The synthetic agonist α,β-meATP is selective for P2X3 receptor subunit and can, therefore, activate heteromeric P2X2/3 receptors as well [48–50] with their characteristic slow plateau current. While colocalization of P2X2 and P2X3 subunits has been previously reported in a number of mouse TG neurons, the contribution by P2X2/3 receptors to membrane currents is relatively small in view of the fact that slow-desensitizing responses are only detected in a minority of these cells [51]. Their small number precluded a systematic evaluation of BNP/NPR-A inhibition on heteromeric P2X2/3 receptors.

The process responsible for the enhanced currents seen after suppressing the BNP/NPR-A system with either anantin or siBNP remains incompletely understood. One contribution was likely to originate from the delayed onset of P2X3 receptor desensitization, an important parameter that controls current amplitude and pain signaling [15]. In the present work monitoring the dynamics of the anantin action showed that BNP/NPR-A system regulated P2X3 receptors almost in all-or-none fashion because there was no gradual modulation of P2X3 currents: in fact, 30 min anantin application was insufficient to affect P2X3 currents, whereas 1 h treatment produced P2X3 receptor upregulation as strong as the one after 24 h, yet these effects were fully reversible on washout.

Because the effects of BNP/NPR-A system inactivation largely resembled the action of CGRP, a well-known positive P2X3 receptor modulator [9], we wondered if the BNP/NPR-A pathway might act by opposing the facilitatory role of CGRP [9, 35–38]. However, simultaneous block of NPR-A and CGRP receptors did not prevent full expression of the potentiating action by anantin, suggesting independence of the BNP/NPR-A system from ambient CGRP.

### BNP signaling relies on P2X3 receptor compartmentalization

Our former studies have reported higher peak amplitudes and slower desensitization time of P2X3 receptors when they are preferentially localized to lipid raft membrane compartments [34], a result similar to what we detected after anantin treatment. The lipid raft structure is highly mobile [52, 53], and potentially adaptable to accommodate redistribution of P2X3 receptors on the time scale seen after inactivation of the BNP/NPR-A system. In accordance with this notion, after anantin or siBNP, the number of P2X3 receptors localized to lipid raft membranes increased significantly without changes in the global receptor level, and this redistribution was completely abolished by disrupting lipid rafts with MβCD. It is, however, noteworthy that, even after application of MβCD (that had made P2X3 currents smaller), anantin or siBNP could retain a moderate degree of enhancing activity. Since the total number of P2X3 receptors had not changed, it is suggested that enhancement of P2X3 amplitudes could be partly, but not entirely, explained by the preferential P2X3 receptors localization to lipid raft membrane fraction. This proposal implied the existence of an additional mechanism, apart from receptor redistribution, employed by the BNP/NPR-A system to regulate P2X3 receptor function.

### Changes in P2X3 phosphorylation state evoked by BNP/NPR-A block

As phosphorylation/dephosphorylation is one of the most common ways of altering receptor functions, for P2X3 receptors such a crucial regulatory role is often played by serine and tyrosine residues [5, 12, 13, 54, 55]. In our experiments anantin application or siBNP significantly reduced the amount of P2X3 serine phosphorylation (with no change in tyrosine phosphorylation), a phenomenon shown before to associated to larger P2X3 currents [13]. Disrupting lipid rafts with MβCD did not affect pSer or its decline after anantin application, suggesting that membrane compartmentalization and receptor phosphorylation were distinct phenomena.

Our working hypothesis is that there are two distinct branches of the BNP/NPR-A pathway targeting P2X3 receptors. One regulates receptor distribution between lipid rafts and non-raft membrane compartments, the other one controls P2X3 serine phosphorylation level. Further investigations are required to unravel the details of the molecular cascade starting from NPR-A activation and resulting in depressed P2X3 currents.

### Intracellular pathways linking NPR-A receptors to P2X3 receptors

In search for intermediate steps of the P2X3 modulating NPR-A pathway, we examined the involvement of kinases such as Cdk5 and CaMKII, and of the phosphatase calcineurin, all previously shown to play a role in P2X3 modulation [13, 40]. However, experiments with CaMKII or calcineurin antagonists, or siCdk5 indicated that these enzymes did not play a significant role in the NPR-A modulation of P2X3 pSer or current size.

Notwithstanding the elucidation of the intermediate factors controlling P2X3 receptors via NPR-A activity, our study examined the contribution by PKG as a downstream step after BNP/NPR-A activation. Our previous work has reported early changes in cGMP levels evoked by BNP [26] that would be expected to activate PKG, a family of intracellular kinases that regulate multiple intracellular targets [56–58]. Because KT 5823, used to inhibit PKG, mirrored the effects of anantin or siBNP, it seems that PKG activity was, indeed, a critical gateway to modulate P2X3 receptors. Using the ScanProsite tool (http://prosite.expasy.org/cgi-bin/prosite/ScanView.cgi?scanfile=432156126103.scan.gz), we could not identify apparent consensus sites for the kinase activity on mouse P2X3 receptors. It appears, therefore, unlikely that PKG would directly phosphorylate discrete sites in the intracellular domains of the P2X3 receptors.

**Fig. 9:**
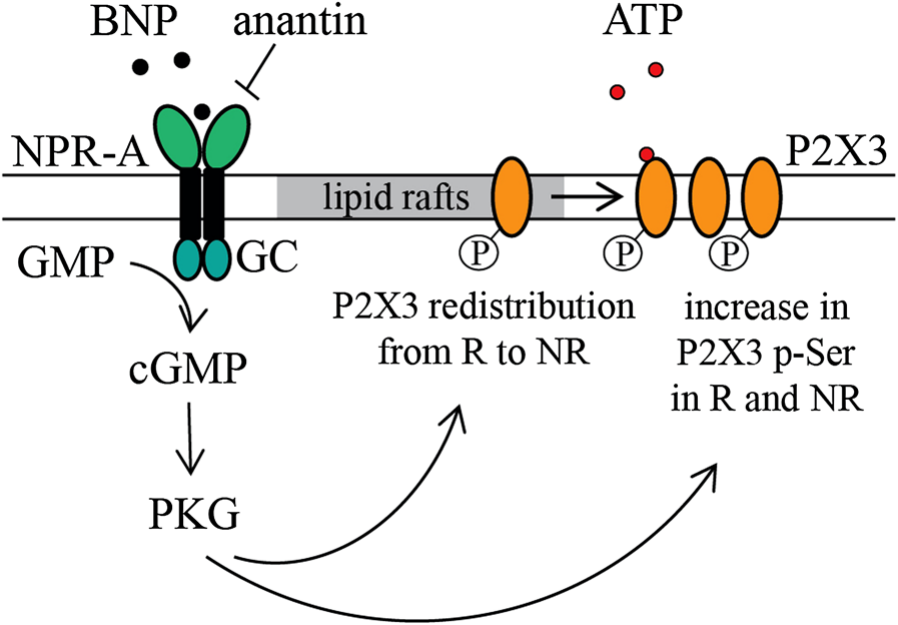
The scheme of BNP/NPR-A dependent downregulation of P2X3 receptors. ATP (*open circles, red*) and BNP (*filled circles*) are extracellular ligands for P2X3 and NPR-A receptors, respectively. P2X3 receptors are localized to lipid raft (*R*) and non-raft (*NR*) membrane compartments. When P2X3 receptors are in *NR*, they show less efficient signaling. BNP by activating NPR-A receptors of sensory neurons activates guanylyn cyclase (*GC*) to increase intracellular cGMP and activate PKG. The latter indirectly regulates, in a negative fashion, P2X3 function by determining their localization to the non-raft domains and by facilitating their pSer

Figure 9 illustrates our proposal that the operation of P2X3 receptors under normal conditions is controlled by the constitutively active NPR-A pathway due to ambient BNP. In our diagram, the basal level of BNP, which is partly produced in TG and mainly supplied to the tissue with the blood flow [24], constantly downregulates P2X3 receptors, elevating their agonist threshold [26]. Hence, BNP via PKG activation mediated by NPR-A receptors would help to localize P2X3 receptors to non-lipid raft membrane compartments, to control their serine phosphorylation state, and to speed up their desensitization without impairing the ability to respond to the natural agonist ATP.

While our scheme includes a P2X3 inhibitory role of BNP via guanylate cyclase and cGMP production, any interaction between P2X3 receptors and other guanylate cyclase activators like nitric oxide (NO) remains unclear. NO donors such as glyceryl trinitrate (GTN) are used as models to induce trigeminal pain and delayed migraine [59]. Former studies show that GTN is effective only when given systemically [60] and that its migraine-provoking activity is dependent on CGRP [61, 62]. This process is, however, incompletely understood because CGRP antagonists do not block GTN headache [63]. Since activation of P2X3 receptors is known to trigger membrane translocation of the NO synthetic enzyme and NO production [64], it is possible to speculate that P2X3 receptor activity is an upstream event to NO-evoked cGMP synthesis and pain [65]. Thus, ongoing P2X3 inhibition by BNP via neuronal NPR-A receptors might be a primary mechanism for controlling the threshold to trigeminal nociception regulated by a complex mix of soluble factors.

The inhibitory role of the BNP/NPR-A system via PKG activity appears to be present also in DRG in which BNP (when co-applied with glutamate) depresses the excitability of a subgroup of sensory neurons in a model of inflammatory pain [27]. In this study, BNP reduced the firing frequency of DRG neurons via intracellular signaling leading to activation of large-conductance Ca^2+^-activated K^+^ channels (BK_Ca_ channels). Future investigations are necessary to explore whether P2X3 receptors, that are expressed by 40 % DRG neurons [66], are also a target for the inhibitory action by BNP.

## Conclusions

In conclusion, the present data outline BNP as the first inhibitory neuropeptide to constitutively depress the P2X3 receptor function. The process appears to be NPR-A and PKG dependent and it involves P2X3 serine phosphorylation and receptor redistribution to the non-raft membrane compartments (Fig. 9). This suggestion will have to be studied in the framework of complex mechanisms responsible for acute or chronic nociception at trigeminal sensory ganglion level. In particular, pharmacological or genetic interference with the BNP/NPRA system in vivo and its effects on allodynia or hyperalgesia would significantly enhance the impact of the current results.

## Methods

### Animals and TG primary culture preparations

All animal procedures were conducted in accordance with the guidelines of the Italian Animal Welfare Act and regulations of animal welfare. All treatment protocols were approved by the Scuola Internazionale Superiore di Studi Avanzati (SISSA) ethics committee and are in accordance with the European Union guidelines. Trigeminal ganglion primary cultures were obtained from animals at the age of P12-14 as described previously [51] and employed after 24 or 48 h from plating.

### Chemicals and treatments

A range of validated chemicals were used to uncover molecular mechanisms underlying the main biochemical and electrophysiological observations. The specific NPR-A receptor antagonist anantin (US Biologicals, Salem, MA, USA), the CGRP receptor antagonist CGRP 8-37 (Sigma-Aldrich, Milan, Italy), and the cholesterol depleting agent methyl-β-cyclodextrin (MβCD, Sigma) were dissolved in sterile water and applied to the cell cultures medium. Anantin was applied for 30 min–24 h at the concentration of 500 nM to ensure full block of NPR-A receptors [26, 67]. CGRP 8–37 was applied in 1 μM concentration to effectively block CGRP receptors [39, 68–70]. MβCD (10 mM) was applied for 30 min in accordance with previous reports [34, 71, 72]. We also applied 5 μM FK-506 (dissolved in DMSO), a well-known immunosuppressant drug that induces persistent pain in humans [73, 74] and acts by inhibiting the Ca^2+^-dependent phosphatase calcineurin [75]. KN-93 (5 μM) was used to block the activity of calcium calmodulin mediated kinase II (CaMKII) in accordance with previous studies [40]. KT 5823 (1 μM; Sigma) **w**as used to inhibit PKG [46, 47].

### BNP and cyclin-dependent kinase 5 (Cdk5) silencing

Cultured mouse trigeminal neurons were transfected with specific siRNA BNP (siBNP) oligonucleotides (Sigma), or siCdk5 oligonucleotide (ThermoFisher Scientific, Milan, Italy) using the DharmaFECT transfection reagent (Dharmacon, Lafayette, CO, USA). As control (“scramble”), cells were transfected with siGLO RISC-Free siRNA (Dharmacon). The BNP siRNA and control siRNA were transfected at a final concentration of 100 nM for 24 h in triplicate for each treatment. At 48 h post-transfection, BNP knockdown was confirmed by immunostaining and Elisa assay. A pool of two different oligonucleotide sequences were used to knockdown BNP (NM_008726) expression, as previously published [76]: BNP siRNA-1: sense 5′-CCCAGAGACAGCUCUUGAATT-3′ with antisense 5′-UUCAAGAGCUGUCUCUGGGTT-3′, and BNP siRNA-2: sense 5′-GGCACAAGAUAGACCGGAUTT-3′ with antisense 5′-AUCCGGUCUAUCUUGUGCCTT-3′. To knockdown Cdk5 expression (NM_007668.3), siCdk5 or control siRNA sequences were transfected at a final concentration of 100 nM. Twenty-four hours after silencing, cells were used for protein expression and patch clamping experiments. Efficiency of Cdk5 silencing was tested with Western blot.

### Immunocytochemistry

Forty-eight hours after siRNA-treatment, primary cultures from P12-P14 mouse TG were fixed in 4 % paraformaldehyde and immunofluorescence was carried out as previously described [26]. Polyclonal antibody against BNP (G-011-23; 1:100) was from Phoenix Pharmaceuticals (Burlingame, CA, USA). Secondary antibody conjugated with Alexa Fluor-594 was purchased from Invitrogen (1:500; Milan, Italy). Nuclei were counterstained with DAPI (Sigma). Images were obtained using a Leica TCS SP2 confocal microscope (Wetzlar, Germany).

### Membrane fractionation and protein extraction

Total membrane protein extraction and Triton X-100-soluble and -insoluble fractionation were performed as reported by Gnanasekaran et al. 2011 [34]. To ensure equal loading for each cell lysate, protein extracts were quantified with bicinchoninic acid (BCA; Sigma). The amount of loaded proteins was in the 20–50 µg/ml range. Immunoprecipitation of P2X3 receptors from membrane fractions (raft and non-raft) was performed as previously reported [12].

### Western blot

Western blot analysis was performed according to the methods previously reported [12, 26]. Cells were lysed in ODG buffer solution (2 % *n*-octyl-beta-d-glucopyranoside, containing 1 % Nonidet P-40, 10 mM Tris pH 7.5, 150 mM NaCl, plus protease inhibitors mixture; Complete, Roche Applied Science, Basel, Switzerland). Proteins were separated in 10 % SDS–polyacrylamide gel and transferred to nitrocellulose membranes. Immunoblotting was performed with the following validated primary antibodies: rabbit anti-P2X3 (1:1000; Santa Cruz Biotechnology, Heidelberg, Germany) rabbit anti-NPR-A (1:1000, Abcam), anti-β-tubulin III (1:2000; Sigma), mouse anti-β-actin (1:3000, Sigma), mouse anti-flotillin 1 (1:250; BD Biosciences), anti-phospho-Serine (1:500, millipore), anti-phospho-Tyrosine (1:500, millipore) [26, 34, 40]. Signals were revealed after incubation with recommended secondary antibodies conjugated with horseradish peroxidase by using ECL detection reagent (Amersham Biosciences, Piscataway, NJ, USA) and recorded by the digital imaging system Alliance 4.7 (UVITEC, Cambridge, UK).

### ELISA assay for BNP

Samples from TG cultures and their culture medium were collected 24 h after plating and prepared as described by Vilotti et al. (2013). The BNP levels were assessed using an ELISA kit (Abnova, Hidelberg, Germany), following the instructions of the manufacturer. Sample BNP levels were determined using standard concentration curves and evaluated in duplicate. Data were normalized to sample protein concentration, as determined by the BCA method (Sigma).

### Electrophysiology

Electrophysiology experiments were performed using previously reported protocols [39, 51]. Cultured TG neurons were patch-clamped in the whole-cell configuration under continuous perfusion (3 mL/min) with physiological solution containing (in mM): 152 NaCl, 5 KCl, 1 MgCl_2_, 2 CaCl_2_, 10 glucose, and 10 HEPES (pH adjusted to 7.4 with NaOH). Patch pipettes were filled with the following solution (in mM): 140 KCl, 0.5 CaCl_2_, 2 MgCl_2_, 2 Mg_2_ATP3, 2 GTP, 10 HEPES and 10 EGTA (pH adjusted to 7.2 with KOH) and had a resistance of 3–4 MΩ. Recordings were performed from small and medium size trigeminal neurons [39] held at −65 mV after correction for liquid junction potential. Currents were filtered at 1 kHz and acquired by means of a DigiData 1200 interface and pClamp8.2 software (Molecular Devices, Sunnyvale, CA, USA). In accordance with our previous studies α,β-methylene-adenosine-5′-triphosphate (α,β-meATP; Sigma), a stable synthetic agonist of P2X3 receptors [33] was applied for 2 s at a standard dose of 10 µM [26, 33, 39] using a fast superfusion system (Rapid Solution Changer RSC-200; BioLogic Science Instruments, Claix, France) to evoke reproducible near-maximal P2X3 responses [13, 77]. Data were collected from at least 4 individual experiments with number of cells equal or greater than 25. Responses were measured as peak amplitudes and presented as current density values (pA/pF) after normalization to the cells’ capacity, in order to eliminate differences in currents originated from cell size variation. The only exception were experiments with MβCD, where the data were shown as peak current amplitudes, since MβCD, as a cholesterol depleting agent, is expected to change cell capacitance.

### Statistical analysis

Data are expressed as mean ± standard deviation (s.d.) or as mean ± standard error of the mean (SEM), where *n* indicates the number of independent experiments or the number of investigated cells, as indicated in figure legends. Statistical analysis was performed using nonparametric Mann–Whitney rank sum test, or the Student’s *t* test, after the software-directed choice of non-parametric or parametric data, respectively (Matlab; Sigma Plot and Sigma Stat Software, Chicago, IL, USA), or Kruskal–Wallis test for multiple comparison. A p value of <0.05 was accepted as indicative of a statistically significant difference.

## Abbreviations

α,β-meATP: α,β-MethyleneATP
BCA: Bicinchoninic acid
s.d.: Standard deviation
BNP: Brain natriuretic peptide
CaMKII: Calcium calmodulin mediated kinase II
CGRP: Calcitonin gene-related peptide
DRG: Dorsal root ganglia
GTN: Glyceryl trinitrate
MβCD: Methyl-β-cyclodextrin
NO: Nitric oxide
NPR-A: Natriuretic peptide receptor-A
PKG: CGMP-dependent protein kinase
pSer: Serine phosphorylation
pTyr: Tyrosine phosphorylation
sem: Standard error of the mean
TG: Trigeminal ganglion
